# Hybrid Caffeic Acid-Based DHFR Inhibitors as Novel Antimicrobial and Anticancer Agents

**DOI:** 10.3390/antibiotics13060479

**Published:** 2024-05-23

**Authors:** Renu Sehrawat, Ritu Pasrija, Priyanka Rathee, Deepika Kumari, Anurag Khatkar, Esra Küpeli Akkol, Eduardo Sobarzo-Sánchez

**Affiliations:** 1 Department of Pharmaceutical Sciences, Maharshi Dayanand University, Rohtak 124001, India; rsehrawat29@gmail.com; 2 Department of Biochemistry, Maharshi Dayanand University, Rohtak 124001, India; ritupasrija.biochem@mdurohtak.ac.in (R.P.); kalirawandeepika@gmail.com (D.K.); 3 Faculty of Pharmaceutical Sciences, Baba Mastnath University, Rohtak 124021, India; priyankapharmacy@bmu.ac.in; 4 Department of Pharmacognosy, Faculty of Pharmacy, Gazi University, Ankara 06330, Türkiye; 5 Instituto de Investigación y Postgrado, Facultad de Ciencias de la Salud, Universidad Central de Chile, Lord Cochrane 417, Santiago 8330507, Chile; eduardo.sobarzo@ucentral.cl; 6 Department of Organic Chemistry, Faculty of Pharmacy, University of Santiago de Compostela, 15782 Santiago de Compostela, Spain

**Keywords:** caffeic acid, anticancer, antimicrobial, DHFR inhibitor, in silico design

## Abstract

A novel series of 1,2,4-triazole analogues of caffeic acid was designed, synthesized, characterized, and assessed for their capacity to inhibit DHFR, as well as their anticancer and antimicrobial properties. A molecular docking analysis was conducted on DHFR, utilizing PDB IDs 1U72 and 2W9S, aiming to design anticancer and antimicrobial drugs, respectively. Among all the synthesized derivatives, compound CTh7 demonstrated the highest potency as a DHFR inhibitor, with an IC_50_ value of 0.15 μM. Additionally, it exhibited significant cytotoxic properties, with an IC_50_ value of 8.53 µM. The molecular docking analysis of the CTh7 compound revealed that it forms strong interactions with key residues of *homo sapiens* DHFR such as Glu30, Phe34, Tyr121, Ile16, Val115, and Phe31 within the target protein binding site and displayed excellent docking scores and binding energy (−9.9; −70.38 kcal/mol). Additionally, synthesized compounds were screened for antimicrobial properties, revealing significant antimicrobial potential against bacterial strains and moderate effects against fungal strains. Specifically, compound CTh3 exhibited notable antibacterial efficacy against *Staphylococcus aureus* (MIC = 5 µM). Similarly, compound CTh4 demonstrated significant antibacterial activity against both *Escherichia coli* and *Pseudomonas aeruginosa*, with MIC values of 5 µM for each. A docking analysis of the most active antimicrobial compound CTh3 revealed that it forms hydrogen bonds with Thr121 and Asn18, a π–cation bond with Phe92, and a salt bridge with the polar residue Asp27.

## 1. Introduction

Cancer encompasses a vast array of diseases in which abnormal cells proliferate uncontrollably, originating from various areas within the body and potentially spreading to nearby or distant regions [1]. It ranks among the primary causes of mortality worldwide, and its occurrence is steadily rising. As per a World Health Organization (WHO) report, it is a significant factor contributing to mortality that led to approximately 10 million deaths in the year 2020, accounting for roughly one in every six fatalities [2]. Overall, the development of novel anticancer drugs is crucial for improving cancer treatment options, enhancing patient recovery, overcoming resistance, and addressing the unique challenges associated with different types of cancer. Antimicrobial resistance (AMR) is also a growing health concern that poses a threat to effectively treat an increasing range of microbial infections. The WHO has acknowledged AMR as one of the ten major global public health challenges, and about 10 million deaths each year by 2050 could be caused by drug-resistant diseases. In response, the WHO has categorized resistant bacteria into different groups based on their priority. Priority 1 includes critically important bacteria like *Acinetobacter baumannii* and *Pseudomonas aeruginosa*, which are associated with severe healthcare-associated infections. Priority 2 consists of highly important bacteria such as *Staphylococcus aureus* and *Helicobacter pylori*, responsible for common infections. Priority 3 includes moderately important bacteria like *Streptococcus pneumoniae* and *Haemophilus influenzae*, associated with community-acquired infections [3,4,5]. The emergence of multidrug-resistant (MDR) pathogens has severely compromised the efficacy of existing antimicrobial drugs. Current chemotherapeutic agents are also associated with severe side effects and resistance. Hence, *Dihydrofolate reductase* (DHFR; E.C. 1.5.1.3) emerges as a compelling target as it is a ubiquitous enzyme found in the cells of nearly all living organisms. It plays a crucial role in converting 7,8-dihydrofolate into 5,6,7,8-tetrahydrofolate by using the NADPH coenzyme in this process. Tetrahydrofolate is crucial in the synthesis of essential building blocks of DNA and important amino acids by the de novo process [6,7,8]. The inhibition of this enzyme leads to a deficiency of the active form of folic acid, which, in turn, disturbs nucleotide biosynthesis and, ultimately, leads to cell death. This trait is used to inhibit cancerous cells and microbial growth [9,10,11,12,13]. DHFR inhibitors have been utilized for many years in treating both cancer and microbial infections. However, there is still room for exploring new lead compounds for DHFR inhibition activity due to the development of resistance against existing DHFR inhibitors. Methotrexate, an established antimetabolite, is extensively used as a major chemotherapeutic agent. Nevertheless, methotrexate also faces resistance in mammalian cancer cells. It could occur through various mechanisms. These include a reduced methotrexate uptake by the reduced folate carrier (RFC), increased methotrexate efflux facilitated by over-expression ATP-binding cassette (ABC) transporters, decreased methotrexate polyglutamation by *Folypolyglutamate synthetase*, elevated expression of DHFR protein via DHFR gene amplification, reduced methotrexate affinity resulting from DHFR mutation, and combinations of these mechanisms [14,15,16]. Resistance to trimethoprim has indeed been documented, attributed to various mechanisms, including the activation of alternative metabolic pathways and the overproduction of resistant chromosomal DHFR enzymes [17]. However, the persistent resistance to existing DHFR inhibitors and the absence of novel DHFR inhibitors in the pipeline for decades have compelled us to conduct concentrated research in this area.

To identify a novel structural class for DHFR inhibition, caffeic acid was selected as a lead to design novel derivatives as in our previous investigation aimed at identifying novel compounds from natural sources that could inhibit DHFR; caffeic acid demonstrated significant activity and favorable binding scores [18]. Although numerous studies have been conducted to assess the antimicrobial and anticancer properties of caffeic acid, the lack of understanding regarding its mechanism of action and its interaction with DHFR has significantly impeded the utilization of caffeic acid as a lead and the discovery of promising novel derivatives based on caffeic acid [19,20,21,22,23]. Screening various heterocyclic compounds for the development of novel derivatives of caffeic acid, we found that compounds containing nitrogen and oxygen atoms have demonstrated significant biological efficacy [24]. Extensive studies have shown that 1,2,4-triazole analogues, depending on their substituents, possess diverse biological properties such as antimicrobial, analgesic [25,26,27], anti-tumor [28,29], anti-inflammatory [30], anti-hypertensive [31], and antiviral [32] activities. The 1,2,4-triazole moiety is present in numerous standard medicines, emphasizing its significance [33]. Likewise, Schiff bases have garnered interest in the medicinal field owing to their extensive range of biological activities, including antimicrobial [34,35,36], antitubercular [37,38], anti-HIV, anticancer [39], anti-inflammatory, analgesic [40], and anticonvulsant properties [41]. In the meantime, nitrogen and sulfur small-ring heterocycles have been regarded as appealing foundational structures for an extended period because of their ease of synthesis, versatility, and relevance in therapeutics [42]. By considering the notable significance of Schiff bases, caffeic acid, and 1,2,4-triazole in the fields of biology and medicine, we have designed and synthesized hybrid caffeic acid-based derivatives containing Schiff bases derived from 1,2,4-triazole and evaluated their potential as bioactive compounds. A library of analogues was designed by using different aldehydes with aliphatic, aromatic, and heterocyclic rings with different substitutions. This approach aimed to inspect the influence of the substituent’s and ring size on the effectiveness of compounds. Through computational docking studies, we have identified the most promising compounds for further analysis.

Based on the observations outlined, we hereby present the design and synthesis of novel molecular scaffolds containing 1,2,4-triazole rings using caffeic acid as a synthon in the hope of obtaining medicinally significant DHFR inhibitors as antimicrobial and anticancer agents. The compounds were characterized using data from the FTIR, NMR, and elemental analysis. Their DHFR inhibition, antibacterial, antifungal, and anticancer capabilities were examined against specific standard strains and cell line.

## 2. Results and Discussion

### 2.1. Molecular Modeling

Structure-based drug design (SBDD) was utilized to design novel compounds and assess their affinities towards a target protein. This approach is widely accepted for discovering novel leads and eliminating incompatible derivatives [43]. A molecular docking analysis was performed for all designed derivatives of caffeic acid, targeting the three-dimensional coordinates of *homo sapiens* DHFR (PDB ID 1U72) co-crystallized with methotrexate (MTX). Additionally, for microbial infection, the analysis included the S. aureus trimethoprim-resistant variant S1DHFR (PDB ID: 2W9S) [44]. Based on our previous research, caffeic acid, a lead compound, was selected as it exhibited favorable interactions with the oxidative enzyme DHFR [18]. Consequently, modifications were made at the carboxylic acid group of caffeic acid to design ligands, and a 1,2,4-triazole ring was introduced to mimic the pyrimidine ring of trimethoprim. DHFR inhibition activity has also been reported for the 1,2,4-triazole ring [13,45]. The necessity of the free phenolic hydroxyl group in caffeic acid arises from its ability to establish hydrogen bonds with crucial amino acid residues in the target protein. The designed derivatives were subjected to virtual screening using the Schrödinger suite of molecular modeling software 13.1. Ligands exhibiting better and comparable binding energy and docking scores than caffeic acid, standard trimethoprim, and methotrexate were retained for further exploration of the mechanistic approach toward DHFR. The well-docked compounds were subsequently synthesized and evaluated for their DHFR inhibitory potential, as well as their anticancer and antimicrobial properties. The most active compounds were further investigated to understand their mechanistic interactions within the binding site.

#### 2.1.1. Molecular Docking Interaction Analysis of *Homo Sapiens* DHFR (PDB ID 1U72)

**Figure 1:**
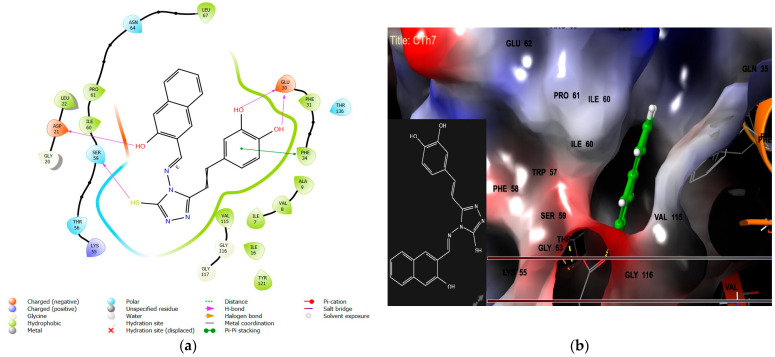
(**a**) 2D model illustrating the interaction between compound CTh7 and active site amino acids, highlighting various types of bonds. (**b**) A three-dimensional representation of CTh7 within the active pocket of the DHFR (PDB ID 1U72) receptor, highlighting amino acid residues in close proximity to the active site.

**Figure 2:**
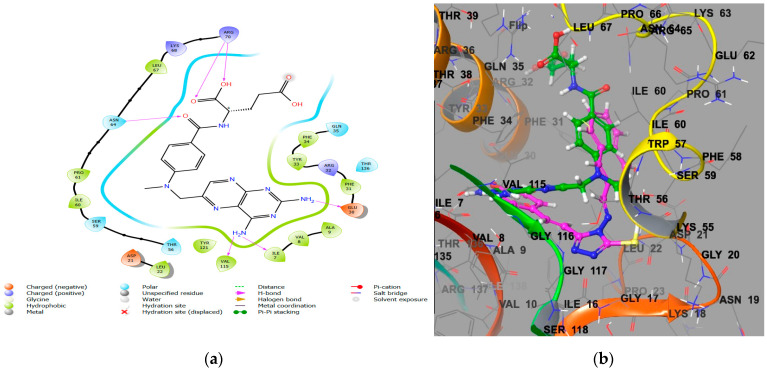
(**a**) Depicts a 2D interaction perspective of methotrexate with PDB ID 1U72. (**b**) Superimposed image of CTh7 (magenta) and MTX (green) in the binding pocket of the DHFR (1U72) receptor with amino acid residues present in proximity of the active site.

A molecular docking analysis was performed to design caffeic acid derivatives using the structure of homo sapiens DHFR (PDB ID 1U72), co-crystallized with methotrexate as a protein target. Among all the designed derivatives, compound CTh7 exhibited a similar docking performance to methotrexate, with a binding energy of −70.38 kcal/mol and a docking score of −9.9. It is bound to the same site, interacting with key residues such as Glu30, Ser59, and Asp21 by hydrogen bonding and with Phe34 by π–π stacking, as shown in Figure 1. This finding aligns with M. Wang’s 2017 results [46]. It also snugly fits into the hydrophobic pocket of the modeled protein. These findings were further corroborated by cross-docking investigations involving the target protein, ligand, and MTX. The most effective anticancer agent CTh7 with MTX may be seen in the superimposed image (Figure 2), revealing similar pharmacophores and occupying the same active site regions within the binding pocket of DHFR. Methotrexate, a standard inhibitor of DHFR, was also tested against PDB ID 1U72. It forms hydrogen bonds with Glu30, Ile7, Val115, Asn64, and Arg70 (Table 1). Redocking the co-crystallized ligands was performed to validate the docking protocol, and the RMSD value was found to be 1.19, which was satisfactory for approving the docking protocol. The molecular docking analysis revealed that nearly all the synthesized ligands engaged the same core of the target enzyme, surrounded by residues such as Leu67, Pro61, Ile60, Tyr121, Ile16, Val115, Leu22, Ile7, Val8, Ala9, Phe31, and Phe34. To validate the docking results, selected designed ligands were subsequently synthesized in the laboratory and assessed for their anticancer and DHFR inhibitory activities.

#### Molecular Dynamics Simulation Studies

**Figure 3:**
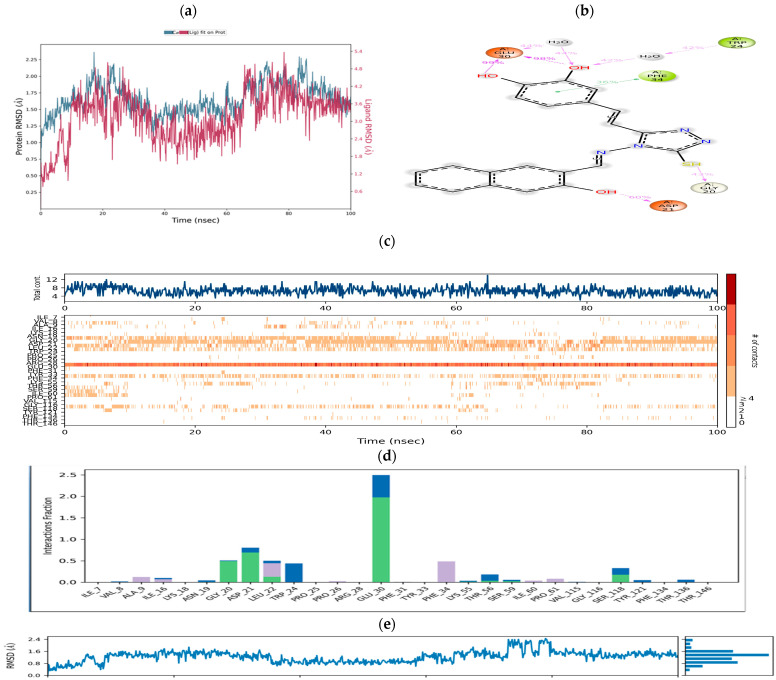
(**a**) RMSD relative to the initially minimized complex of the molecule during MD simulations {protein (PDB ID 1U72): left *Y*-axis and ligand CTh7: right *Y*-axis}. (**b**) A schematic of detailed ligand CTh7 atom interactions with the protein residues (interactions that occur more than **30.0%** of the simulation time in the selected trajectory). (**c**) A timeline representation of the interactions and contacts between protein and ligand CTh7. (**d**) Protein interaction with ligand (green: hydrogen bond; purple: hydrophobic; blue: water bridges). (**e**) Root mean square deviation of a ligand with respect to the reference conformation.

The stability of the docked pose of the most active DHFR inhibitor (CTh7) in the *homo sapiens*-DHFR binding site (PDB ID: 1U72) was assessed through a molecular dynamics simulation. The RMSD (root mean square deviation) of both the protein backbone and heavy atoms over the entire 100 ns MD simulation was carried out. The results indicated that the compound CTh7 exhibited a remarkable stability within the active site of the enzyme. The study further highlighted significant interactions between CTh7 and crucial amino acids such as Glu30, Trp24, Asp21, Gly20, and Phe34. Throughout the simulation, CTh7 consistently maintained interactions with the active site residues of the protein (Figure 3). The Ligand RMSD (shown on the right *Y*-axis in Figure 3a) provided insights into the stability of the ligand in the binding pocket of the protein. The ‘Lig Fit Prot’ shows the RMSD of a ligand when the protein–ligand complex is first aligned on the protein backbone of the reference, and then the RMSD of the ligand heavy atoms is measured. Notably, RMSD values remained below 2 Å for the first 10 ns after reaching equilibrium, confirming the conformational stability of the protein–ligand (CTh7) complex.

#### 2.1.2. Molecular Docking Interaction Analysis of Microbe DHFR (PDB ID 2W9S)

**Figure 4:**
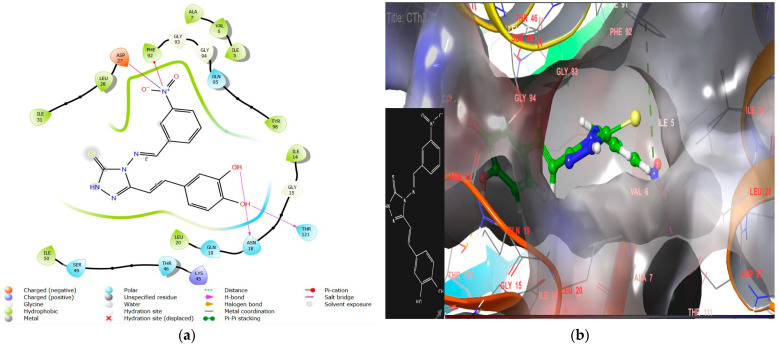
(**a**) 2D model illustrating the interaction between compound CTh3 and active site amino acids, highlighting various types of bonds. (**b**) A three-dimensional representation of CTh3 within the active pocket of the DHFR (2W9S) receptor, highlighting amino acid residues in close proximity to the active site.

The molecular docking technique was utilized to design caffeic acid derivatives, utilizing the *S. aureus* trimethoprim-resistant variant S1DHFR (PDB ID: 2W9S), co-crystallized with TMP (trimethoprim) as the protein target [44]. Among all the designed derivatives, the docking pose of the highly effective antibacterial compound CTh3 revealed hydrogen bonds forming between the phenolic hydroxyl groups of the compound and the Asn18 and Thr121 residues of the target protein. The nitro group of the compound formed a π–cation bond with Phe92 and a salt bridge with the polar (negatively charged) residue Asp27, as shown in Figure 4. This type of interaction resembled that of the co-crystallized ligand TMP. The compound was observed to be firmly positioned within the binding site of the enzyme and engaging in hydrophobic interactions with residues Tyr98, Ile50, Ile31, Leu28, Ala7, Val6, Ile5, Phe92, Leu20, and Ile14, resulting in excellent docking scores and binding energies (−9.8, −71.12 kcal/mol). 

**Figure 5:**
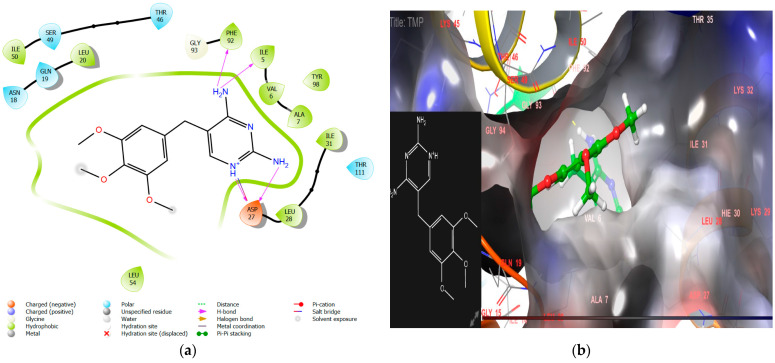
(**a**) 2D model illustrating the interaction between trimethoprim and binding site amino acids, highlighting various types of bonds. (**b**) A three-dimensional representation of TMP within the active pocket of the DHFR (2W9S) receptor, highlighting amino acid residues in close proximity to the active site.

These desirable types of interactions might explain the superior antimicrobial activity of CTh3. Figure 5 demonstrates the various interactions between the co-crystallized ligand trimethoprim and the target protein. These interactions include hydrogen bond formation with Ile5, Phe92, and Asp27 residues and salt bridge formation with Asp27. 

**Figure 6:**
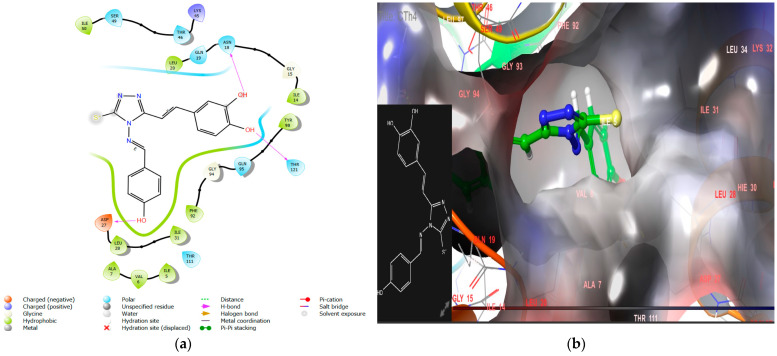
(**a**) 2D model illustrating the interaction between compound CTh4 and active site amino acids, highlighting various types of bonds. (**b**) A three-dimensional representation of CTh4 within the active pocket of the DHFR (2W9S) receptor, highlighting amino acid residues in close proximity to the active site.

Another ligand, CTh4, exhibited remarkable interactions within the target protein, forming hydrogen bonds with key residues Asp27, Asn18, and Thr121, as shown in Figure 6. The phenolic group of the ligand and the triazole moiety are embedded in a hydrophobic cavity, leading to excellent docking scores and binding energies (−9.3, −72.84 kcal/mol).

**Figure 7:**
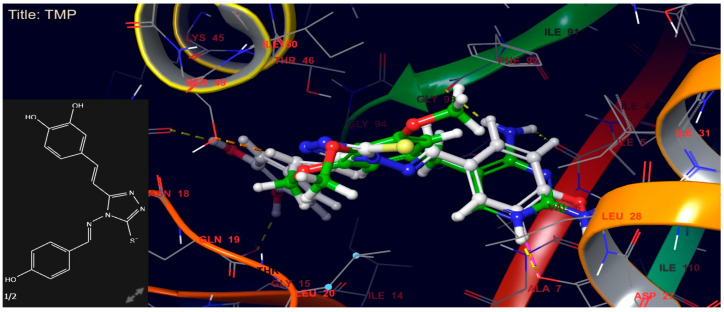
Superimposed image of CTh4 (green) and TMP (white) in the pocket of the DHFR (2W9S) receptor active site with amino acid residues present in proximity to the active site.

Hydrophobic interactions were also seen between the ligand and hydrophobic amino acid residues, securely holding the compound CTh4 within the compact substrate cavity (superimposed image of CTh4 and TMPs shown in Figure 7). 

**Figure 8:**
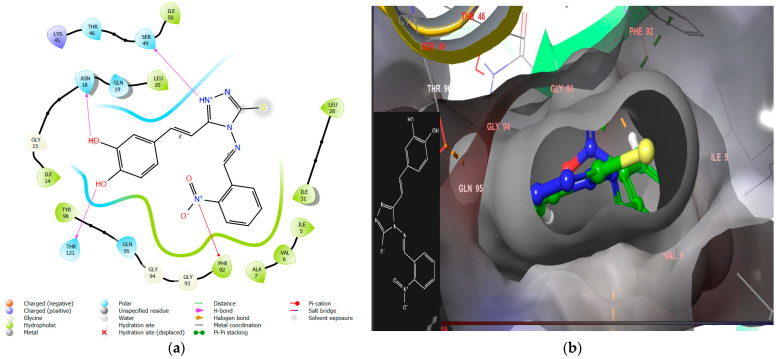
(**a**) 2D model illustrating the interaction between compound CTh6 and active site amino acids, highlighting various types of bonds. (**b**) Three-dimensional representation of CTh6 within the active pocket of the DHFR (2W9S) receptor, highlighting amino acid residues in close proximity to the active site.

Compound CTh6 exhibited promising docking outcomes, having a docking score of −9.4 and a binding energy of −73.77 kcal/mol (Figure 8). The molecular docking analysis revealed that nearly all tested ligands occupied the same core of enzymes, surrounded by residues such as Asp 27, Leu 54, Ile 50, Phe 92, Tyr 98, Ile 14, Leu 20, Trp 22, Leu 28, Tyr 109, Ala 7, Val 6, Ile 5, Ile 31, Leu 20, Gly 93, Gly 94, Gln 19, Asn 18, Thr 121, and Thr 46 (additional information given in Table 2). These findings are further supported by the results of Ikram et al. and Adnane Aouidate [47,48]. The redocking of the co-crystallized ligand had been performed to confirm the docking protocol, resulting in a satisfactory RMSD value of 0.56 Å.

#### 2.1.3. ADMET Evaluation

ADMET prediction plays a crucial role in drug development by enabling the rapid and preliminary testing of ADMET properties before compounds undergo an in vitro evaluation. The evaluation of ADMET properties revealed that the new compounds exhibit excellent human intestinal absorption, as predicted by Caco-2 permeability, MDCK permeability, P-glycoprotein inhibition (Pgp), and HIA values (Table 3). This advantage is attributed to the superior lipophilicity of the compounds, which facilitates their passage through biological membranes. Human oral bioavailability predictions (F20%) indicate a good oral bioavailability for all compounds except CTh4, CTh7, and CTh. The distribution, as evaluated by PPB permeability, indicated significant plasma protein binding for all compounds except compound CTh. This finding highlights the need for tailoring compounds to improve distribution. However, the distribution assessed by VD and BBB for the novel synthesized compounds was found to be excellent. An analysis revealed that the compounds could inhibit most cytochrome P450 family isoenzymes involved in drug metabolism, particularly cytochrome 2D6 and 2C19. Excretion, evaluated in terms of clearance, highlighted that the novel derivatives exhibit excellent total clearance. Toxicity, the last parameter studied in the ADMET profile, revealed a critical drawback concerning the positive probability of AMES toxicity for the derivatives, indicating potential mutagenicity and carcinogenicity. Additionally, compounds CTh7 and CTh10 showed positive results for skin sensitivity, and compound CTh10 exhibited a respiratory toxicity probability. However, all other synthesized compounds were free from rat oral acute toxicity, respiratory toxicity, and skin sensitization. All derivatives are devoid of the probability of cardiotoxicity, indicating no inhibitory effect on hERG.

### 2.2. Chemistry

**Scheme 1:**
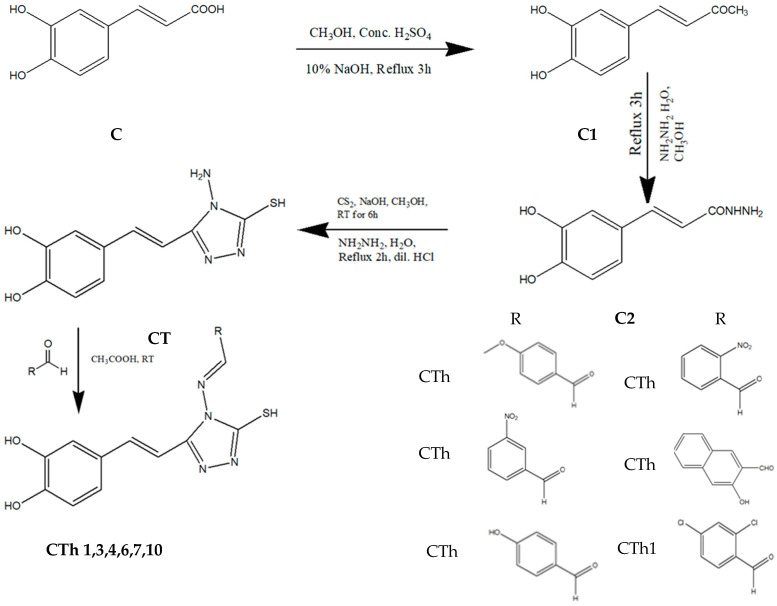
General synthetic scheme to synthesize caffeic acid derivatives.

The in silico screening revealed that the introduction of a 1,2,4-triazole ring to caffeic acid could enhance the interaction between the derivatives and the DHFR receptor. To further improve the interaction and activity, hundreds of Schiff bases of 4-[2-(4-amino-5-mercapto-4H-triazol-3-yl)vinyl]benzene-1,2-diol were designed. These derivatives were synthesized following a multistep process outlined in Scheme 1 [45]. 

The process involved substitution, hydrazinolysis, and condensation reactions. Starting with the compound 4-(3,4-dihydroxyphenyl)but-3-en-2-one (C1), which was prepared through a Fischer–Speier esterification reaction in methanol, it was refluxed with hydrazine hydrate to obtain 3-(3,4-dihydroxyphenyl)acrylohydrazide (C2). The compound C2 underwent cyclization to form 4-[2-(4-amino-5-mercapto-4H-triazol-3-yl)vinyl]benzene-1,2-diol (CTh). Various analogues of the 1,2,4-triazole were synthesized by reacting CTh with different aldehydes and utilizing acetic acid as a catalyst. A single-spot TLC was used to confirm that the reaction had been completed successfully. The structures of the synthesized derivatives were elucidated by using ^1^H NMR, ^13^C NMR, FTIR, and elemental analysis. In the IR spectra of all the synthesized compounds, distinctive peaks were observed, signifying the existence of N-H stretching (3100–3300 cm^−1^), phenolic OH stretching (3600–3400 cm^−1^), aromatic C-H stretching (3000–2900 cm^−1^), S-H stretching (2350–2361 cm^−1^), C-S bending (740–760 cm^−1^), aromatic ring vibrations (1796–1700 cm^−1^), and -C=N stretching (1520–1590 cm^−1^). These observations suggest the formation of the triazole ring and Schiff bases in the synthesized compounds. A band near 1600 cm^−1^ was observed in all synthesized derivatives, which highlights the formation of the imine bond C=N of the Schiff base [49]. The absence of a broad band of carboxylic acid between 3200–2500 also indicates the conversion of caffeic acid into a product. The formation of these compounds is additionally substantiated by the ^1^H NMR signals, as evidenced by the absence of the carboxylic acid peak at around δ 12 throughout every compound that was synthesized.

#### Characterization of Synthesized Compounds

*4-(2-(4-(4-methoxybenzylideneamino)-5-mercapto-4H-1,2,4-triazol-3-yl)vinyl)benzene-1,2-diol* (**CTh1**). M.Wt.: 368.41; Yield: 72%; M.P.: 220–221; R_f_: 0.67; FTIR in cm^−1^: 3612 (O-H stretching, phenol), 3116 (C-H stretching, aromatic), 2825 (C-H stretching, aliphatic), 1197 (C-O-C stretching, methoxy), 2358 (S-H stretching), 1654 (C=N stretching, triazole), 817 (C-S bending); ^1^H NMR (DMSO-d6, 400 MHz) in δ: 9.88 (s, 1H; Schiff base; imine), 8.66 (s, IH; OH),8.64 (s, 1H; OH)), 7.88 (d, *J =* 8.8 Hz, 1H; Ar-H), 7.82 (d, *J =* 8.7 Hz, 1H; Ar-H), 7.14 (d, *J =* 8.7 Hz, 2H; Ethylene-H), 7.06 (d, *J =* 8.7 Hz, 1H; Ar-H), 6.76–6.69 (m, 1H; Ar-H), 3.87 (s, 3H; OCH3), 3.83 (s, 1H; SH); ^13^C NMR (DMSO-d6) in δ: 191.81(Ar-C; 2C), 164.67(imine-C; 1C), 161.00(Ar-C; 1C), 133.71(Ar-C; 3C), 131.81(Het Ar-C; 2C), 130.45(ethylene-C; 1C), 130.09(ethylene-C; 1C), 114.98(Ar-C; 3C), 114.86(Ar-C; 1C), 114.26(Ar-C; 1C), 56.16(methyl-C; 1C), 56.00(methyl-C; 1C, S), Anal calculated for C_18_H_16_N_4_O_3_S: C, 58.68; H, 4.38; N, 15.21; O, 13.03; S, 8.70 Found: C, 58.69; H, 4.39; N, 15.20; O, 13.04; S, 8.69.

*4-(2-(4-(3-nitrobenzylideneamino)-5-mercapto-4H-1,2,4-triazol-3-yl)vinyl)benzene-1,2-diol* (**CTh3**). M.Wt.: 383.38; Yield: 75%; M.P.: 232–233; R_f_: 0.66; FTIR in cm^−1^: 3617 (O-H stretching, phenol), 3114 (C-H stretching, aromatic), 2926 (C-H stretching, aliphatic), 2358 (S-H stretching), 1525 (C=N stretching, triazole), 698 (C-S bending); ^1^H NMR (DMSO-d6, 400 MHz) in δ: 8.72 (s, 1H; Schiff base; imine), 8.47 (s, 1H; OH), 8.47 (s, 1H; OH), 7.84 (d, *J =* 8.6 Hz, 1H; Ar-H), 7.74 (d, *J =* 8.4 Hz, 3H; ethylene-H), 7.67–7.56 (m, 1H; Ar-H), 6.64 (m, 2H; Ar-H), 5.70 (s, 1H; Ar-H), 3.74 (dd, *J =* 7.1 Hz, 1H; Ar-H), 2.89 (s, 1H; SH); 13C NMR (DMSO-d6) in δ: 175.86(Ar-C; 1C), 162.77(imine-C; 1C), 161.03(Het Ar-C; 2C), 148.76–148.73(Ar-C; 1C), 134.91(Ar-C; 1C), 133.76(Ar-C; 1C), 133.64(Ar-C; 1C), 131.51(Ar-C; 1C), 131.11(ethylene-C;1C), 130.85(ethylene-C;1C), 130.64(0C, S), 126.30(Ar-C; 1C), 123.97(Ar-C; 1C), 123.11(0C, S), 121.82(Ar-C; 1C), 119.23(Ar-C; 1C), 115.92(Ar-C; 1C); Anal calculated for C_17_H_13_N_5_O_4_S: C, 53.12; H, 3.67; N, 18.22; O, 16.65; S, 8.34 Found: C, 53.10; H, 3.68; N, 18.23; O, 16.66; S, 8.35. 

*4-(2-(4-(4-hydroxylbenzylideneamino)-5-mercapto-4H-1,2,4-triazol-3-yl)vinyl)benzene-1,2-diol* (**CTh4**). M.Wt.: 354.08; Yield: 77%; M.P.: 219–220; R_f_: 0.59; FTIR in cm^−1^: 3613 (O-H stretching, phenol), 3082 (C-H stretching, aromatic), 2360 (S-H stretching), 1620 (C=N stretching, triazole), 730 (C-S bending); ^1^H NMR (DMSO-d6, 400 MHz) in δ: δ 8.91 (s, 1H; Schiff base; imine), 8.70 (s, 2H; OH), 8.57 (d, *J =* 7.7 Hz, 1H; Ar-H), 8.37 (d, *J =* 8.3 Hz, 1H; Ar-H), 8.32 (d, *J =* 7.7 Hz, 2H; ethylene-H), 7.82 (t, *J =* 8.0 Hz, 3H; Ar-H), 2.89 (s, 1H; SH); ^13^C NMR (DMSO-d6) in δ: 161.03(imine-C; 1C), 148.76–148.73 (Het Ar-C; 2C, S), 148.63(Ar-C; 2C), 135.67 (Ar-C; 2C), 134.91 (Ar-C; 1C), 133.76–133.64 (Ar-C; 3C), 131.51(ethylene-C; 1C), 131.11 (ethylene-C; 1C), 130.74 (Ar-C; 1C), 126.30(Ar-C; 1C), 124.77(Ar-C; 1C), 124.15 (Ar-C; 1C); Anal calculated for C_17_H_14_N_4_O_3_S: C, 57.62; N, 15.81; H, 3.98; O, 13.54; S, 9.05 Found: C, 57.64; N, 15.82; H, 3.99; O, 13.53; S, 9.04

*4-(2-(4-(2-nitrobenzylideneamino)-5-mercapto-4H-1,2,4-triazol-3-yl)vinyl)benzene-1,2-diol* (**CTh6**). M.Wt.: 383.38; Yield: 72%; M.P.: 216–217; R_f_: 0.6; FTIR in cm^−1^: 3615 (O-H stretching, phenol), 3112 (C-H stretching, aromatic), 2359 (S-H stretching), 1652 (C=N stretching, triazole), 732 (C-S bending), 1525 (NO_2_ stretching); ^1^H NMR (DMSO-d6, 400 MHz) in δ: 8.92 (s, 1H; Schiff base; imine), 8.71 (s, 2H; OH), 8.41–8.29 (m, 3H; Ar-H), 7.82 (d, *J =* 7.6 Hz, 2H; Ar-H), 2.73 (s, 1H; SH); ^13^C NMR (DMSO-d6) in δ: 160.95 (imine-C; 1C), 148.82–148.66 (Het Ar-C; 2C), 135.70(Ar-C; 2C), 134.90(Ar-C; 2C), 131.11(ethylene-C; 3C), 130.73(Ar-C; 1C), 126.28(Ar-C; 2C), 124.77(Ar-C; 1C), 123.11(Ar-C; 3C); Anal calculated for C_17_H_13_N_5_O_4_S: C, 53.12; H, 3.67; N, 18.22; O, 16.65; S, 8.34 Found: C, 53.13; H, 3.69; N, 18.23; O, 16.66; S, 8.35. 

*4-(2-(4-(4-naphthalen-2-ol)-5-mercapto-4H-1,2,4-triazol-3-yl)vinyl)benzene-1,2-diol* (**CTh7**). M.Wt.: 404.44; Yield: 72%; M.P.: 201–202; R_f_: 0.55; FTIR in cm^−1^: 3613 (O-H stretching, phenol), 3115 (C-H stretching, aromatic), 2929 (C-H stretching, aliphatic), 2358 (S-H stretching), 1621 (C=N stretching, triazole), 784 (C-S bending); ^1^H NMR (DMSO-d6, 400 MHz) in δ: 10.01 (s, 1H; Schiff base; imine), 8.67 (d, *J =* 8.6 Hz, 1H; Ar-H), 8.61 (s, 1H-Ar -H), 8.47 (s, 1H; Ar -H), 8.05 (d, *J =* 9.0 Hz, 1H; ethylene-H), 7.93 (dd, *J =* 8.0, 1.4 Hz, 1H; naphthalene- H), 7.63 (dd, *J =* 8.4, 6.8, 1.5 Hz, 1H; naphthalene- H), 7.57 (dd, *J =* 8.5, 6.8, 1.5 Hz, 1H; naphthalene- H), 5.70 (s, 3H; OH), 5.29 (d, *J =* 2.8 Hz, 1H; Ar -H), 4.50 (s, 1H; SH); ^13^C NMR (DMSO-d6) in δ: 162.77(Ar-C; 1C), 161.03(imine-C; 1C), 148.76–148.63(Het Ar-C; 2C), 135.67(Ar-C; 2C), 134.91(Ar-C; 1C), 133.76(Ar-C; 1C), 133.64(Ar-C; 1C), 131.51(ethylene-C; 1C), 131.11(ethylene-C; 1C), 130.74(Ar-C; 1C), 130.64(Ar-C; 1C), 126.30(Ar-C; 1C), 124.77(Ar-C; 1C), 124.15(Ar-C; 1C), 123.97(Ar-C; 1C), 123.11(Ar-C; 3C), 121.82(Ar-C; 1C); Anal calculated for C_21_H_16_N4O_3_S: C, 62.36; N, 13.85; H, 3.99; O, 11.87; S, 7.93 Found: C, 62.37; N, 13.84; H, 4.00; O, 11.87; S, 7.94. 

*4-(2-(4-((2,4-dichlorocyclohexa-1,5-dienyl)methyleneamino)-5-mercapto-4H-1,2,4-triazol-3-yl)vinyl)benzene-1,2-diol* (**CTh10**). M.Wt.: 409.28; Yield: 72%; M.P.: 222–223; R_f_: 0.62; FTIR in cm^−1^: 3741 (O-H stretching, phenol), 3114 (C-H stretching, aromatic), 2924 (C-H stretching, aliphatic), 2361 (S-H stretching), 767 (C-S bending), 1651 (C=N stretching, triazole), 1521 (C=C stretching, aromatic), 767 (C-Cl aryl stretching); ^1^H NMR (DMSO-d6, 400 MHz) in δ: 10.28 (s, 2H; OH), 7.89 (s, 1H; Schiff base; imine), 7.88–7.85 (m, 2H; Ar-H), 7.73 (t, *J =* 2.5 Hz, 1H; Ar-H), 7.65 (d, *J =* 1.9 Hz, 1H; Ar-H), 7.62 (d, *J =* 2.1 Hz, 1H; ethylene-H), 7.57 (dd, *J =* 2.3 Hz, 1H; Ar-H), 6.69 (d, *J =* 2.4 Hz, 1H; Ar-H), 4.10–4.04 (m, 1H; Ar-H), 3.77–3.67 (m, 4H; Ar-H), 2.55 (s, 1H; SH); 13C NMR (DMSO-d6) in δ: 189.37(Ar-C; 3C), 172.61 (imine-C; 1C), 147.97(Ar-C; 1C), 140.21(Het Ar-C; 1C), 137.62(Het Ar-C; 1C), 131.55(Ar-C; 1C), 131.42(ethylene-C; 2C), 130.77(Ar-C; 1C), 128.78(Ar-C; 1C), 115.83(Ar-C; 1C), 61.62(1C, S), 60.59(Ar-C; 1C), 60.45(Ar-C; 1C), 56.07–56.00(Ar-C; 2C); Anal calculated for C_17_H_14_C_l2_N4O_2_S: C, 49.89; H, 3.45; N, 13.69; O, 7.82; S, 7.83 Found: C, 49.91; H, 3.46; N, 13.70; O, 7.82; S, 7.84. 

*4-(2-amino 1-mercapto-4H-1,2,4-triazol-3-yl)vinyl)benzene-1,2-diol* (**CTh**). M.Wt.: 250.28; Yield: 89%; M.P.: 235–236; R_f_: 0.5; FTIR in cm^−1^: 2925 (C-H stretching, aromatic), 2855 (C-H stretching, aliphatic), 3198 (N-H stretching, amine), 2360 (S-H stretching), 1680 (C=N stretching, triazole), 769 (C-S bending); ^1^H NMR (DMSO-d6, 400 MHz) in δ: 8.72 (s, 2H; OH), 8.47 (s, 1H; Ar-H), 7.84 (d, *J =* 8.6 Hz, 2H; ethylene-H), 7.74 (d, *J =* 8.4 Hz, 2H; Ar-H), 5.70 (s, 2H; NH2), 3.75 (s, 1H; SH); ^13^C NMR (DMSO-d6) in δ: 161.03 (Het Ar-C; 1C), 148.63(Ar-C; 2C), 135.67 (Het Ar-C; 1C, S), 134.91 (Ar-C; 1C), 131.11 (ethylene-C; 2C), 126.30 (Ar-C; 1C), 124.77 (Ar-C; 1C), 123.11(Ar-C; 1C); Anal calculated for C_10_H_10_N_4_O_2_S: C, 47.99; N, 22.39; H, 4.03; O, 1.79; S, 12.81; Found: C, 48.01; N, 22.39; H, 4.05; O, 1.78; S, 12.82.

### 2.3. Biological Activity

#### 2.3.1. DHFR Inhibition Assay

To evaluate the efficacy, mechanism, and confirmation of the results obtained from molecular docking, the in vitro inhibitory effect of the synthetic derivatives on DHFR was assessed. This assessment was carried out using the DHFR assay kit (catalog number CS0340, Sigma-Aldrich, Burlington, MA, USA) by using a spectrophotometric method. The enzymatic degradation of dihydrofolic acid to tetrahydrofolic acid was monitored at 340 nm for 180 s, following the procedure recommended by the manufacturer of the assay kit. IC_50_ values of the synthesized derivatives were calculated and presented in Table 2. The obtained in vitro activity results aligned with the computational predictions, with a few minor deviations. Methotrexate, an established standard for DHFR inhibition, was included in the screening process under identical conditions for comparison purposes and as a positive control. The results demonstrated the significant potency of the studied compounds in inhibiting the target enzyme at micromolar concentrations. Among all the synthesized derivatives, the most potent lead compound was CTh7, with an IC_50_ value of 0.15 µM. It might be due to the fact that it forms hydrogen bonding with key amino acid residues such as Glu30, Ser59, and Asp21, and π–π stacking with Phe34 against the modeled *homo sapiens* DHFR. Compound CTh4 also demonstrated a remarkable inhibition against DHFR, with an IC_50_ value of 2.04 µM. As the IC_50_ range for Schiff bases of 4-[2-(4-amino-5-mercapto-4H-triazol-3-yl)vinyl]benzene-1,2-diol fell between 0.15 and 17.03 µM, these findings suggest that DHFR may be the molecular target of these analogues, potentially exerting an antimetabolite effect. These compounds exhibit strong DHFR inhibitory activity, which is consistent with their cytotoxic and antibacterial properties.

#### 2.3.2. Assessment of Anticancer Activity

The anticancer potential of synthesized derivatives was assessed by targeting the MCF-7 breast cancer cell line. The results, summarized in Table 1, revealed that compound CTh7 exhibited remarkable anticancer activity, with an IC_50_ value of 8.53 μM, but less potent than the standard drug doxorubicin (IC_50_ = 3.62 μM). This outcome is in line with the findings from docking studies and DHFR inhibition assays, suggesting that CTh7 might act as a potent DHFR inhibitor. These results provide evidence that caffeic derivatives can be effectively designed to function as DHFR inhibitors, which is a promising direction for anticancer drug development. However, a further evaluation on normal cells is necessary in order to ascertain whether the observed effects are truly selective towards cancer cells and not simply indicative of the general toxicity of the compounds. Overall, this research sheds an important light on the potential of this novel class of compounds as anticancer agents, opening avenues for future studies and further optimization to improve their efficacy and therapeutic potential against breast cancer.

#### 2.3.3. Antimicrobial Activity

The minimum concentration required to halt the growth of a micro-organism within 18 to 24 h is referred to as the minimum inhibitory concentration (MIC) [50]. The serial dilution technique was utilized to evaluate the antimicrobial activity of synthesized derivatives against Gram-negative, Gram-positive, and fungal strains. Trimethoprim, ampicillin, tetracycline, fluconazole, and voriconazole were used as reference drugs. To dissolve the reference and sample derivatives, DMSO was employed as a solvent. The synthesized compounds were evaluated for their antibacterial effectiveness against *E. coli*, *P. aeruginosa*, and *S. aureus*, and their antifungal potential was tested against *Candida albicans* and *Aspergillus niger*. Based on the results of the MIC values presented in Table 4, it was evident that compound CTh3 displayed a remarkable efficacy against *S. aureus* with an MIC value of 5 µM, surpassing the performance of trimethoprim (63 µM) and ampicillin (11 µM). Compound CTh6 also demonstrated excellent activity against *S. aureus*, exhibiting an MIC value of 10 µM. Additionally, compound CTh4 exhibited notable effectiveness against *P. aeruginosa*, *E. coli*, and *C. albicans*, with MIC values of 5 µM, 5 µM, and 11 µM, respectively, outperforming the reference drugs. The synthesized compounds showed significant antibacterial activity, comparable to or even better than standard drugs such as trimethoprim and ampicillin. However, their inhibitory effects against *C. albicans* and *A. niger* were relatively lower compared to their antibacterial activity, with only CTh3, CTh4, and CTh10 showing marginal effectiveness. Compound CTh3 exhibited an MIC of 20 µM against *C. albicans*, while compound CTh10 displayed the highest efficacy against *A. niger* with an MIC of 39 µM. *E. coli* proved to be the most sensitive to all synthesized compounds, and *A. niger* was the least sensitive. Overall, the antibacterial activity was influenced by the scaffold type and the substituent in the aromatic moiety. It is concluded from this research that Schiff bases containing electron-withdrawing groups such as -NO_2_ and -OH on their aromatic ring exhibit significant antimicrobial activity. These groups are found to interact through various bonds, such as hydrogen bonding, π–cation interactions, and salt bridge formations with essential amino acid residues of DHFR, which might contribute to their effectiveness against micro-organisms.

## 3. Structure–Activity Relationship

To study the correlation between biological activity and the structures of newly synthesized caffeic acid derivatives, an SAR analysis was performed, and the findings are given below:

- The introduction of nitro substitution at the ortho and meta positions of an aromatic ring has been found to enhance both DHFR inhibitory activity and antimicrobial activity in derivatives. For instance, compounds CTh3 and CTh6 exhibit good DHFR inhibitory and excellent antimicrobial activity, with CTh3 showing an MIC of 5 µM against *S. aureus*.
- Incorporating a hydroxyl group, as seen in CTh4, significantly increases the DHFR inhibitory activity, likely due to the hydrogen bond formation with the essential negatively charged amino acid residue Asp27. This compound also demonstrates excellent antimicrobial activity with MICs of 5 µM, 5 µM, and 11 µM against *E. coli*, *P. aeruginosa*, and *C. albicans*, respectively.
- Furthermore, when a chloro group is substituted on the aromatic ring of a Schiff base, the antifungal activity is further enhanced, particularly against *A. niger*. For example, compound CTh10 exhibits strong activity against *A. niger* with an MIC of 39 µM.
- The introduction of a bulky aromatic group leads to a sharp increase in DHFR inhibitory activity and remarkable anticancer activity. Compound CTh7, for example, displays an IC_50_ value of 8.53 µM against the MCF-7 cell line.

**Figure 9:**
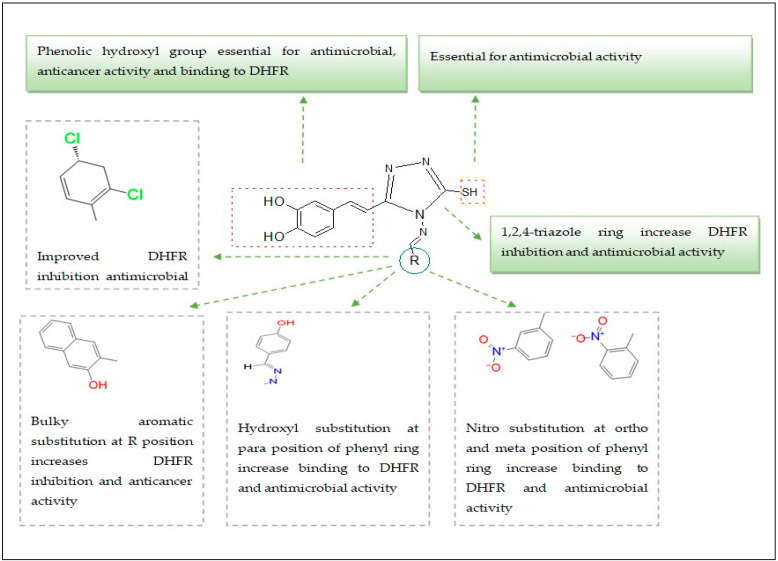
Explication of SAR of newly synthesized caffeic acid derivatives.

Upon analyzing the biological activities, it can be concluded that the occurrence of a phenolic hydroxyl group in caffeic acid is essential for DHFR inhibitory activity. Additionally, introducing a triazole ring on the carboxylic functional group of the acid further enhances antimicrobial and anticancer activity. The Schiff base formation with the triazole ring significantly improves biological activity. Substituting electronegative groups like nitro, hydroxyl, and chloro groups enhances antimicrobial activity remarkably. The incorporation of bulky groups in Schiff bases may increase the DHFR inhibition and anticancer potential against the MCF-7 tumor cell line, possibly due to the increased lipophilic character of the compound. Overall, these findings suggest that specific substitutions and modifications can significantly impact the biological activities of the compounds under study, as summarized in Figure 9.

## 4. Materials and Methods

### 4.1. Instruments and Chemicals

The research employed analytical-grade chemicals and reagents, which were obtained from Merck (Darmstadt, Germany), Sigma-Aldrich (Burlington, MA, USA), Loba Chemie (Mumbai, India), and CDH (New Delhi, India). The progression of the reactions was monitored on pre-coated TLC plate (60 GF 254, silica gel 0.25 mm; Merck, Germany). Dichloromethane, methanol, and acetic acid in a ratio of 9.4:0.5:0.1 mL, respectively, served as the developing solvent system, and visualization of spots was carried out under a UV lamp. The melting point of synthesized compounds was determined utilizing the open capillary method using a sonar melting point apparatus, and the reported values were uncorrected. The infrared spectra of the synthesized compounds were recorded using an FTIR Bruker ATR instrument (Vertex 70v; Bruker Optik GmbH, Rosenheim, Germany) in cm^−1^. The ^1^H NMR and ^13^C NMR spectra were acquired using a Bruker Avance II 400 NMR (Bruker, Wissembourg, France) spectrophotometer operating at frequencies of 400 MHz and 100 MHz, respectively. DMSO-d6 was used as the solvent, while tetramethylsilane was utilized as the internal standard. Chemical shifts were recorded in δ unit. Additionally, elemental analysis was conducted using the Perkin–Elmer 2400 Series II CHNS-O analyzers. For the in vitro assessment of DHFR inhibitory activity, a DHFR assay kit (Sigma-Aldrich, catalog number CS0340) was used.

### 4.2. Molecular Modelling

#### 4.2.1. Molecular Docking

A molecular docking investigation was conducted to check enzyme–ligand interaction using Maestro Glide software 13.1 (New York, NY, USA) [51]. X-ray crystal structures of DHFR were retrieved from the RCSB Protein Data Bank with PDB IDs 1U72 for anticancer and 2W9S for antimicrobial drug design [52]. Selections of PDB IDs were carried out on the basis of resolution and species. The caffeic acid derivatives library was built using the Chemdraw Ultra software 12.0, and Maestro’s LigPrep tool was utilized to prepare ligands for energy minimization, correct the co-ordinates, and generate tautomers to obtain appropriate conformation; 32 stereoisomers per ligand were allowed; at target, pH 7 +/− 2 was set as a default option, and the force field was OPLS3e. The protein preparation wizard of Schrödinger was used to prepare proteins to obtain optimized and energy-minimized protein configurations [53,54,55]. DHFR protein is directly downloaded on the Maestro workspace interface from the RCSB Protein Data Bank. The protein preparation involved executing pre-process steps, and the Epik tool was employed to ionize heteroatoms at biological pH, ensuring the maintenance of a biosimilar environment. Following the pre-process, the ultimate step involved obtaining an energy-minimized structure using OPLS3e as the force field. Grid was generated by picking molecules, and docking site (active site) validation was carried out by splitting co-crystallized ligands from the minimized prepared protein complex and then re-docking to the active site. For the 1U72 and 2W9S PDB IDs, the RMSD (root mean square deviation) values were found to be 1.19 and 0.56, respectively, which is very satisfactory for approving the docking protocol. To analyze the interaction of proteins and ligands, docking with extra precision (XP) was used. The most promising ligand was chosen for laboratory synthesis and subsequent assessment of its pharmacological activity.

#### 4.2.2. Binding Free Energies and MM/GBSA

The MM/GBSA energy calculation prime module of Maestro was used to determine the free binding energy of designed derivatives of caffeic acid. This was accomplished using the VSGB solvation model and other default set options for calculating the binding energy. This approach is widely acknowledged and accepted for assessing the free binding energy of designed compounds to proteins. It aids in rationalizing experimental observations and virtual screening outcomes, effectively distinguishing between drugs and binders only [56,57]. The following formula was used to determine the energy differences:

ΔE = Ecomplex − Eligand − Eprotein

#### 4.2.3. ADMET Evaluation

In this research, the ADMETlab 2.0 online tool was used to evaluate various pharmacokinetic parameters and toxicity properties [58]. The ADMET prediction for the synthesized compounds was predicted to ensure adherence to Lipinski’s rules of five, which indicate their drug-like characteristics as designed ligands. The absorption of ligands was assessed by predicting values across various factors, including intestinal absorption, membrane and skin permeability, P-glycoprotein substrate or inhibitor status, and human intestinal absorption (HIA). The drug distribution of ligands was evaluated by predicting parameters such as plasma protein binding (PPB), volume of distribution (VD), and blood–brain barrier permeability (BBB) values. Metabolism predictions were carried out by CYP (cytochrome P450) models for substrate or inhibition, while excretion was assessed using clearance (CL) and drug half-life (T1/2). Toxicity is predicted using various parameters like AMES toxicity, hERG inhibition, rat oral acute toxicity, respiratory toxicity, and skin sensitization.

#### 4.2.4. Molecular Dynamics Simulations

MD simulations of most potential compound was conducted at the binding site of *human*-DHFR (PDB ID: 1U72) using Desmond package within Maestro. The construction of the system involved utilizing the OPLS-AA force field within an explicit solvent, employing the single-point-charge (SPC) water model, denoted as OPLS-AA/SPC, and adding Cl^−^ counter-ions to neutralize the overall charge. The resulting complex system consisted of around 28,215 atoms. Before running equilibration and extensive-production MD simulations, the systems were minimized and pre-equilibrated using Desmond’s default relaxation protocol. The MD simulations ran for 100 ns, employing the NPT ensemble for the integration of equations of motion. The temperature was held at 300 K through the Nosé–Hoover thermostat method with a relaxation time of 1 ps. Trajectory recording occurred at 20 ns intervals throughout the entire MD run [59].

### 4.3. Synthesis of Caffeic Acid Derivatives

#### 4.3.1. Synthesis of 4-(3,4-Dihydroxyphenyl)but-3-en-2-one (C1)

A solution was prepared by dissolving 0.131 mol of caffeic acid in 250 mL of methanol, and then concentrated H_2_SO_4_ (0.131 mol) was added dropwise to the solution (Scheme 1). The resultant solution was refluxed for duration of 3 h. The single-spot TLC technique was utilized to check the completion of the reaction. After reflux, the mixture was allowed to cool to room temperature, and any excess methanol was subsequently distilled off. The resultant product was rendered alkaline by the addition of a 10% NaOH solution in cold water. A brown-colored product identified as C1 was obtained, filtered, and subsequently washed with cold water. To purify the product further, it was recrystallized from a 1:1 mixture of methanol and water.

#### 4.3.2. Synthesis of 3-(3,4-Dihroxyphenyl)acrylohydrazide (C2)

A solution was prepared by dissolving 0.078 mol of compound C1 in a mixture of hydrazine hydrate (0.233 mol) in methanol (50 mL). The resulting solution was then refluxed for a duration of 3 h. A solid, sticky, brown-colored residue was obtained, identified as C2. The end of the reaction was verified by conducting a single-spot TLC. The product was filtered by suction filtration, followed by washing with methanol, and, ultimately, dried.

#### 4.3.3. Synthesis of 4-[2-(4-Amino-5-mercapto-4H-triazol-3-yl)vinyl] benzene-1,2-diol (CTh)

Carbon disulfide (0.031 mol) and compound C2 were added to a methanol solution of NaOH (0.031 mol) while stirring continuously. The reaction mixture was stirred at room temperature for a period of 6 h. The precipitates formed during the reaction were filtered using suction, washed with water, and then dried under vacuum. The product obtained from the initial step was directly utilized in the subsequent step without undergoing additional purification. This product was subjected to a treatment with 0.062 mol of hydrazine hydrate and water (40 mL), and the resulting mixture was refluxed for a period of 2 h. During the refluxing, hydrogen sulfide gas evolved, and a light brown homogeneous solution was obtained. The completion of the reaction was verified by conducting a single-spot TLC technique. The resulting solution was diluted with ice-cold water. The solution was subsequently acidified with dilute hydrochloric acid, leading to the formation of light brown precipitates of compound CTh. The precipitates of CTh were filtered, washed, and subsequently recrystallized with alcohol to yield the purified product.

#### 4.3.4. General Synthetic Procedure for the Synthesis of Schiff Bases

Dissolve compound CTh (0.002 mol) in 10 mL of dimethylformamide (DMF). Different aldehydes (0.005 mol) and 2–3 drops of glacial acetic acid (as a catalyst) were added to the solution of compound CTh. Allow the mixture to stir for 4 h at room temperature. After the completion of the reaction, pour the resulting product into crushed ice to induce precipitation. The precipitates so obtained were filtered by suction, rinsed with cold water, and subsequently subjected to recrystallization in alcohol to obtain purified products.

### 4.4. Assessment of Biological Activity

#### 4.4.1. In Vitro DHFR Inhibition Assessment

To evaluate the ability of the synthesized derivatives to block DHFR activity, a spectrophotometric assay technique was utilized at 340 nm for 180 seconds to detect the enzyme-catalyzed conversion of dihydrofolic acid to tetrahydrofolic acid. The DHFR assay kit (Sigma-Aldrich, catalog number CS0340) was used in this assay. The kit was tested using recombinant DHFR, NIH 3T3, A431, and CHO cell lines, as well as rat kidney, brain, liver, and muscle tissue extracts. Dihydrofolic acid, NADPH, standard drug solution, assay buffer, and DHFR stock solutions were prepared according to the instructions given in the technical bulletin that came along with the test kit. Except for the assay buffer, all stock solutions were stored on ice.

Each well of a 96-well clear microplate was filled with a calculated amount of assay buffer (190 μL), followed by 6 μL of diluted DHFR, which was thoroughly mixed. Different concentrations of serially diluted synthesized compounds (0.01–10 µM) and diluted NADPH (1.2 μL) were introduced to the wells. The microplate was then covered with parafilm, and the contents were mixed by inversion. Following the addition of 1 μL of substrate dihydrofolic acid, the absorbance was measured at 340 nm over a period of 180 s using an ELISA reader (Gen BioTek Microplate Reader, Winooski, VT, USA) in kinetic mode at room temperature. Plotting the relationship between absorbance and time generates the slope for each test inhibitor sample. The relative inhibition percentages were calculated using Equation (1) [60,61,62].

% relative inhibition = (slope of [EC] − slope of [S]/slope of [EC]) × 100(1)

where S is the sample of interest, EC (enzyme control).

#### 4.4.2. Anticancer Activity (MTT Assay)

The potential newly synthesized derivatives were evaluated for their cytotoxic activity on the MCF-7 cell line (sourced from NCCS in Pune, India) using the MTT (3-[4,5-dimethylthiazol-2-yl]-2,5-diphenyl tetrazolium bromide) assay. To perform the assay, 10,000 cells were placed in each well of 96-well plates and left to incubate for 24 h in Dulbecco’s Modified Eagle Medium (DMEM). The medium was supplemented with 1% antibiotic solution and 10% FBS (fetal bovine serum), and the incubation occurred at a temperature of 37 °C in an environment containing 5% CO_2_. The next day, the cells were exposed to various concentrations of the derivatives, which had been prepared in an incomplete medium. After a 24 h incubation period, MTT solution (603 µM) was introduced into the cell cultures. The cultures were then subjected to additional 2 h incubation. Upon concluding the experiment, the culture supernatant was extracted, and the cellular matrix was dissolved in 100 µL of DMSO. The absorbance of the resulting solution underwent evaluation using an ELISA plate reader (iMark, Biorad, Hercules, CA, USA) at 540 nm. Triplicate measurements were performed to enhance the reliability and reproducibility of the results obtained [63,64,65,66].

#### 4.4.3. Antimicrobial Activity

The antibacterial and antifungal activity of potential synthesized compounds was evaluated using the broth microdilution method to determine their minimum inhibitory concentration (MIC). All the synthesized derivatives were tested against bacterial strains *S. aureus* (ATCC FDA209-P), *P. aeruginosa* (ATCC 27853), and *E. coli* (ATCC 25922) and fungal strains *A. niger* (MTCC 1688) and *C. albicans* (ATCC SC5314). Individual MIC (µM) values were determined using serial dilution technique with Mueller–Hinton Broth (MHB) media for bacterial growth and Roswell Park Memorial Institute (RPMI) media for fungal growth. Ampicillin, trimethoprim (TMP), tetracycline, voriconazole, and fluconazole were used as reference drugs to set benchmarks. Bacterial strains were cultured in MHB and incubated at 37 ± 1 °C for 24 h, while the fungal strain (*C. albicans*) was grown in RPMI medium at 37 ± 1 °C for 48 h, and the growth of *A. niger* was observed at 25 ± 1 °C for 7 days in the same RPMI medium. The turbidity of the spore suspension was corrected to 0.5 McFarland by adding distilled water. Stock solutions of synthesized compounds were prepared in DMSO solvent and serially diluted to determine the MIC value. Each spore (0.1 mL) was put into each well holding the finally serially diluted synthesized compound concentration, standards, and controls. Optically clear (MIC-0) endpoint criteria, which defined MIC as resulting in 99.9% suppression of visible bacterial and fungal growth following incubation, were used. colony-forming unit (CFU) was also determined by taking 100 µL cultures from the twelfth well, which were dispensed to the tubes containing 900 µL of saline and serially diluted. Agar plate spotting was carried out for each spore and incubated for 24 h at 37 ± 1 °C (for bacteria spores), 48 h at 37 ± 1 °C (for *C. albicans*), and 7 days at 25 ± 1 °C (for *A. niger*) to determine CFU. To assess reproducibility, duplicate tests were performed on three separate days [67,68,69,70].

## 5. Limitation and Future Scope

This research shows promise for the development of a new class of DHFR inhibitors with potential anti-cancer and anti-microbial applications. The synthesized compounds act potently against DHFR in in vitro assays and are predicted to have good drug-like properties. The predicted pharmacokinetic parameters and toxicity data indicate favorable ADME properties, suggesting that the compounds are likely to be well-absorbed, distributed effectively, metabolized efficiently, and excreted without significant toxicity concerns in the tested models. Additional research is imperative in order to validate the efficacy and safety of these compounds in living organisms. Conducting further testing in animal models (in vivo) and eventually progressing to clinical trials is essential to establish their safety profile and efficacy, thereby confirming their status as safe compounds.

Future research can explore these compounds in greater detail. We can investigate their activity in different animal models and test them against other diseases, such as tuberculosis and malaria. Additionally, by incorporating different heterocyclic rings instead of 1,2,4-triazole, we can design and synthesize novel caffeic acid derivatives. This will provide a clearer understanding of the relationship between a structure of compound and its biological activity. Overall, this study identifies a promising new class of DHFR inhibitors that warrants further exploration to identify optimal drug candidates.

## 6. Conclusions

Novel caffeic acid derivatives were designed, synthesized, and characterized, and their potential to inhibit DHFR, along with their antimicrobial and anticancer properties, was evaluated. To mitigate last-stage failures, it is crucial to investigate the initial pharmacokinetic parameters. An ADMET prediction was conducted, which indicates that all the synthesized compounds have promising drug-like characteristics. All the synthesized derivatives exhibited substantial DHFR inhibition, aligning well with the docking results. Among them, CTh7 showcased the highest DHFR inhibitory activity with an IC_50_ value of 0.15 µM. Enzyme inhibition assays confirmed that the newly synthesized compounds exert their action through DHFR-mediated mechanisms. Compound CTh7 demonstrated remarkable anticancer activity against the breast tumor cell line MCF7, boasting an IC_50_ value of 8.53 µM. The most active DHFR inhibitor and anticancer compound, CTh7, displayed excellent docking scores and binding energy (−9.9, −70.38 kcal/mol), outperforming methotrexate. It bound to the same site where MTX binds. It effectively interacted with key residues such as Glu 30, Phe34, Tyr121, Ile16, Val115, and Phe31. The voluminous and hydrophobic structure of CTh7 allowed it to snugly fit into the hydrophobic pocket of the modeled protein. Synthesized compounds displayed significant inhibitory potency against bacterial strains and moderate activity against fungi. Specifically, compounds CTh3, CTh4, and CTh6 demonstrated excellent antibacterial effects, while compound CTh4 also exhibited strong antifungal activity against *C. albicans.* For the most active antimicrobial agent, a CTh3 docking analysis revealed that it forms two hydrogen bonds with Thr 121 and Asn 18, a π–cation bond with Phe 92, and a salt bridge with the polar residue Asp 27. This interaction pattern resembled that of the co-crystallized ligand trimethoprim. CTh3 exhibited excellent docking scores and binding energy (−9.8, −71.12 kcal/mol), surpassing the standard trimethoprim. Overall, this study has discovered a potentially impactful structural class of compounds with antimicrobial and anticancer properties. These compounds could act as pivotal molecules for continued research and development in these respective fields.

## Figures and Tables

**Table 1:** Molecular docking interaction analysis (PDB ID 1U72) and biological assessment of potential anticancer synthesized compounds and standard drugs.

Docking Parameter/Biological Assessment	CTh1	CTh4	CTh6	CTh7	Methotrexate
Docking score	−8.8	−8.2	−8.8	−9.9	−9.8
Binding energy [▲G (kcal/mol)]	−69.80	−60.61	−65.34	−71.38	−73.93
Hydrogen bond	2-hydrogen-bond with Glu30	2-hydrogen-bond with Glu30	2-hydrogen-bond with Glu30	Asp21, Ser59, and 2-hydrogen-bond with Glu30	Glu30, Ile7, Val115, Asn64, and 2-hydrogen-bond with Arg70
Hydrophobic interaction	Pro61, Ile60, Leu22, Tyr121, Ile16, Val115, Phe34, Phe31, Ala9, Val8, Ile7	Phe31, Phe34, Ile7, Val8, Ala9, Val115, Tyr121, Ile16, Le22, Ile60, Pro61	Leu67, Pro61, Ile60, Tyr121, Ile16, Val115, Leu22, Ile7, Val8, Ala9, Phe31, Phe34	Leu67, Pro61, Ile60, Tyr121, Ile16, Val115, Leu22, Ile7, Val8, Ala9, Phe31, Phe34	Leu67, Phe34, Phe31, Tyr33, Ala9, Val8, Ile7, Tyr121, Val115, Leu22, Pro61, Ile60
π–π stacking	Phe34	Phe34	-	Phe34	-
π–cation bond	-	-	Phe34	-	-
Salt bridge	-	-	Lys55	-	-
DHFR Inhibition IC_50_ (µM) ± SD, *n* = 3	17.03 ± 1.10	2.04 ± 0.12	10.54 ± 0.7	0.15 ± 0.02	0.088 ± 0.001
Anticancer IC_50_ (µM), *n* = 3	147.74 ± 11.4	122.17 ± 7.3	89.25 ± 4.17	8.53 ± 0.19	*

(-): No interaction; (*): Not tested.

**Table 2:** Molecular docking interaction analysis (PDB ID 2W9S) and DHFR inhibition assay of potential antimicrobial synthesized compounds and standard drug.

Compound	Docking Score	Binding Energy [▲G (kcal/mol)]	Hydrogen Bond Interaction	Hydrophobic Interaction	Other Interaction	DHFR Inhibition [IC_50_ (µM) ± SD, *n* = 3]
CTh1	−8.2	−45.28	Asn18, Thr121	Ile50, Leu20, Ile14, Tyr98, Phe92, Tyr109, Ile5, Val6, Ala7, Leu28, Ile31	-	17.03 ± 1.10
CTh3	−9.8	−71.12	Asn18, Thr121	Tyr98, Ile50, Ile31, leu 28, Ala7, Val 6, Ile5, Phe 92, Leu20, Ile 14	π–cation with Phe92, salt bridge with Asp27	8.88 ± 0.4
CTh4	−9.5	−72.84	Asn18, Thr 121, Asp 27	Tyr98, Ile50, Ile31, leu 28, Ala7, Val 6, Ile5, Phe 92, Leu20, Ile 14	-	2.04 ± 0.12
CTh6	−9.4	−73.77	Asn18, Thr 121, Ser 49	Tyr98, Ile50, Ile31, Ala7, Val 6, Ile5, Phe 92, Leu20, Ile 14	π–cation Phe92	10.54 ± 0.7
CTh7	−9.6	−59.68	Asp27, Ser 49	Tyr98, Ile50, Ile31, leu 28, Ala7, Val 6, Ile5, Phe 92, Leu20, Ile 14, Trp 22	-	0.15 ± 0.02
CTh10	−9.3	−57.23	Asn 18, Ile5, Thr 121	Tyr98, Ile50, Ile31, leu 28, Ala7, Val 6, Ile5, Phe 92, Leu20, Ile 14	-	11.30 ± 0.92
CTh	−9.2	−61.45	Ala 7, Phe 92, 2-H-bond interaction with Asn18	Tyr98, Ile50, Ile14, Ile31, Ala7, Val 6, Ile5, Phe 92, Leu20, Ile 14	-	16.92 ± 1.2
TMP	−9.3	−70.12	2-H-bond with Asp 27, Phe 92, Ile5	Ala7, Ile 31, Leu 20, Leu28, Phe 92, Tyr 98, Ile 50, Leu 54, Ile 5, and Val 6	Salt bridge with Asp27	15.33 ± 1.9

(-): No interaction.

**Table 3:** ADMET evaluation of synthesized compounds.

Properties	CTh1	CTh3	CTh4	CTh6	CTh7	CTh10	CTh
Lipinski rule	Accepted	Accepted	Accepted	Accepted	Accepted	Accepted	Accepted
Absorption							
Caco-2 permeability	−4.971	−5.046	−5.055	−5.064	−5.12	−4.947	−5.332
MDCK permeability	8.5 × 10^−6^	1.28 × 10^−5^	7.2 × 10^−6^	9.3 × 10^−6^	7.68 × 10^−6^	1.9 × 10^−5^	3.3 × 10^−6^
Pgp-inhibitor	---	---	---	---	---	---	---
HIA	---	---	---	---	---	---	---
F_20%_	--	---	+++	---	+++	---	+++
F_30%_	+++	--	+++	-	+++	---	+++
Distribution							
PPB	97.807%	98.39%	97.023%	98.224%	99.004	99.41%	50.853%
VD	0.455	0.22	0.404	0.273	0.51	0.924	0.365
BBB penetration	---	---	---	---	---	---	---
Metabolism							
CYP1A2 inhibitor	++	++	++	++	++	++	-
CYP1A2 substrate	--	---	---	---	---	---	---
CYP2C19 inhibitor	-	---	--	-	-	--	---
CYP2C9 substrate	+++	---	+++	---	---	--	++
CYP2D6 inhibitor	--	--	-	---	-	--	---
CYP2D6 substrate	++	-	+	-	+	++	-
CYP3A4 inhibitor	++	++	+++	++	++	+	-
CYP3A4 substrate	--	--	--	--	--	--	---
Excretion							
CL	9.676	8.233	10.334	8.609	9.194	8.044	13.595
T_1/2_	0.746	0.738	0.885	0.790	0.711	0.75	0.909
Toxicity							
hERG blockers	---	---	---	---	---	---	---
AMES toxicity	++	+++	+	++	++	+++	+++
Rat oral acute toxicity	---	---	---	---	---	---	---
Skin sensitization	-	-	-	-	++	++	--
Respiratory toxicity	---	---	---	---	---	++	---

The prediction probability values are transformed into six symbols: 0–0.1 (---), 0.1–0.3 (--), 0.3–0.5 (-), 0.5–0.7 (+), 0.7–0.9 (++), and 0.9–1.0 (+++).

**Table 4:** Antimicrobial activity (MIC in µM) of synthesized derivatives.

Compound	MIC (µM)				
	Bacterial Strain			Fungal Strain	
	*S. aureus*	*P. aeruginosa*	*E. coli*	*C. albicans*	*A. niger*
CTh1	43	86	86	173	-
CTh3	5	10	10	20	83
CTh4	22	5	5	11	-
CTh6	10	10	20	41	83
CTh7	19	19	39	-	-
CTh10	19	39	39	39	39
CTh	63	127	255	-	127
Trimethoprim	63	31	31	255	127
Ampicillin	11	22	11	NT	NT
Tetracycline	144	36	36	NT	NT
Fluconazole	NT	NT	NT	0.81	260.12
Voriconazole	NT	NT	NT	0.34	5

NT (Not tested): Ampicillin and tetracycline are antibiotics and were, therefore, not tested against fungal strains; trimethoprim was tested against all strain as it was the co-crystallized ligand of selected PDB ID 2W9S during the design process. Similarly, fluconazole and voriconazole are antifungals and were not tested against bacterial strains. -: (No inhibition observed in the studied range).

## Data Availability

The datasets produced during this study are accessible upon request from the corresponding authors. Additional inquiries can be directed to the corresponding author.
